# Conditioning Perspectives for Primary Immunodeficiency Stem Cell Transplants

**DOI:** 10.3389/fped.2019.00434

**Published:** 2019-11-06

**Authors:** Peter Shaw, Judith Shizuru, Manfred Hoenig, Paul Veys, IEWP-EBMT

**Affiliations:** ^1^Children's Hospital at Westmead, Sydney, NSW, Australia; ^2^Departments of Medicine and Pediatrics, Stanford University, Stanford, CA, United States; ^3^Klinik für Kinder- und Jugendmedizin, Universitätsklinikum Ulm, Ulm, Germany; ^4^Great Ormond Street Hospital for Children NHS Foundation Trust, London, United Kingdom

**Keywords:** conditioning, hematopoietic stem cell transplant (HSCT), chemotherapy, immunotherapy, immunoablation

## Abstract

The majority of children undergoing Hematopoietic Stem cell Transplantation (HSCT) require conditioning therapy to make space and prevent rejection of the donor stem cells. The exception is certain children with Severe Combined immune deficiency, who have limited or no ability to reject the donor graft. Transplant conditioning is associated with significant morbidity and mortality from both direct toxic effects of chemotherapy as well as opportunistic infections associated with profound immunosuppression. The ultimate goal of transplant practice is to achieve sufficient engraftment of donor cells to correct the underlying disease with minimal short- and long-term toxicity to the recipient. Traditional combinations, such as busulfan and cyclophosphamide, achieve a high rate of full donor engraftment, but are associated with significant acute transplant-related-mortality and late effects such as infertility. Less “intensive” approaches, such as combinations of treosulfan or melphalan with fludarabine, are less toxic, but may be associated with rejection or low level chimerism requiring the need for re-transplantation. The major benefit of these novel approaches, however, which we hope will be realized in the decades to come, may be the preservation of fertility. Future approaches look to replace chemotherapy with non-toxic antibody conditioning. The lessons learnt in refining conditioning for HSCT are likely to be equally applicable to gene therapy protocols for the same diseases.

## Key Points

- Conditioning is required for the majority of children with non-malignant diseases.
- Some children with severe combined immune deficiency require little or no conditioning—but this usually only corrects T cell function.
- Mixed chimerism may indicate that re-transplantation may be required.
- Less intensive conditioning is:
  ○ Less toxic.
  ○ Associated with more mixed chimerism and may require further procedures.
  ○ May allow long term preservation of fertility.

## Introduction

With the exception of some children with severe combined immunodeficiencies (SCID, see review on SCID), all patients who undergo allogeneic Hematopoietic Stem cell Transplantation (HSCT) require therapy prior to receipt of the graft. This *conditioning therapy* plays a vital role in allowing engraftment of new Hematopoietic progenitor cells (HPC) in the patient. These new HPC can correct some, or occasionally all, of the manifestations of a non-malignant disease.

## The Need for Conditioning

**Figure 1:**
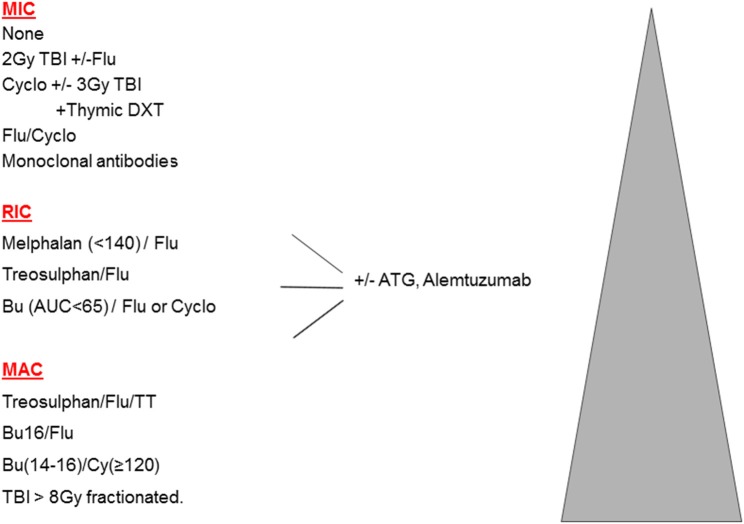
Spectrum of conditioning used.

In the original publications of HSCT for malignant disease, intensive high-dose combinations of irradiation and chemotherapy were used to eradicate resistant leukemia and ablate the bone marrow. These **myeloablative** combinations (MAC) achieved prolonged aplasia, and were associated with full donor chimerism (DC). However, such therapy is associated with a significant burden of early and late toxicities, making MAC less suitable for older patients, or those with significant co-morbidities. This led to the concept of **reduced intensity** (RIC) regimens, which are defined as regimens containing reduced doses of myeloablative drugs (or radiotherapy), which are therefore less likely to achieve marrow ablation and more likely to produce mixed chimerism (MC). For the vast majority of HSCTs, namely older adults with malignant disease, the balance between MAC and RIC is a clear trade off: more transplant–related mortality (TRM) is seen with MAC, and more relapse with RIC; these 2 often counter-balancing each other. Multiple attempts to define RIC in terms of specific drug doses were made during 2006–2009, however, there is a spectrum of conditioning and it is preferable to define truly non-myeloablative or minimally intensive conditioning protocols (MIC) where the initial neutrophil recovery is frequently recipient, MAC protocols which mostly achieve sustained full donor chimerism, and then RIC protocols which comprise all those in-between (1). The spectrum of conditioning is shown in Figure 1.

## Conditioning, Chimerism, and Considerations in Non-malignant Diseases

Full myeloablative conditioning is most likely to achieve full donor chimerism. In non-malignant disease, correction of the underlying disease may be achievable with stable mixed chimerism. It appears that a level of 20–30% donor chimerism in the diseased lineage can achieve correction of the phenotype in, for example, CGD or SCD. But achieving stable mixed donor chimerism reliably with a specific conditioning regimen has been challenging, and 10–20% of patients will require a further procedure such as DLI or 2nd HSCT. This is because patients with non-malignant diseases, who have often had no prior therapy, are more able to reject a graft unless adequate conditioning has been given. In a cohort of over 600 patients with non-malignant disease having unrelated transplants, the cumulative incidence of primary or secondary graft failure at 1 year was 17% (2). Risk factors for graft failure were HLA mismatch and use of RIC.

In contrast, patients with primary immune deficiency (PID), with a partial or complete inability to reject a graft, can achieve MC or full DC with no conditioning or RIC. The first successful allogeneic transplantation reported was performed without conditioning (or graft-versus-host disease prophylaxis) in a patient with X-linked Severe Combined Immunodeficiency (SCID) in 1968 (3). Although engraftment of peripheral T-lymphocytes alone is reliably achieved without conditioning in children with SCID, and this is sufficient to at least transiently allow control of infections and survival, the omission of conditioning also has risks. This was observed in this first patient: by 3 months after administration of the unmanipulated graft from the HLA-identical sibling, the patient developed trilineage aplasia and needed a boost of donor stem cells for hematological reconstitution (4). This graft-versus-marrow (GvM) effect is caused by the donor T-lymphocytes targeting recipient bone marrow cells, leading to a clinical picture of aplastic anemia. This effect is normally hidden by the more obvious marrow depleting effects by chemotherapeutic agents. This effect is more relevant with less intense conditioning regimens, and can be best observed in SCID patients, but is abolished if the graft is also T-cell depleted. Factors that can impact on the outcome of transplant are shown in Table 1.

**Table 1:** Factors that may affect outcome of transplant.

**Relevant factors for therapeutic strategy**	**With an influence on…**
Genetic disease, PID	Ability to reject a graft Cellular lineages to be corrected (myeloid engraftment needed?) Intensity of tolerable conditioning
Clinical condition (infections, organ damage)	Intensity of tolerable conditioning
Donor availability	HLA match rejection GvHD Graft-versus-marrow effect
Stem cell source (bone marrow, PBSCs, CB)	Quantity and quality of cellular (sub-)populations in the graft GvHD Graft-versus-marrow effect
Graft manipulation (*in vitro* or *in-vivo*) T-cell depletion)	GvHD Graft-versus-marrow effect

## Types of Conditioning in Clinical Use

For decades, a combination of myeloablative doses of busulfan (Bu) and cyclophosphamide was used for many children with a wide variety of non-malignant diseases. Its efficacy, in reliably achieving full DC, is not questioned. However, the profile of toxicity, in particular with endothelial injury, most often manifested as veno-occlusive disease (VOD) means we have continued to look for alternatives.

Reducing the Bu dose and increasing the immunosuppression, e.g., by replacing cyclophosphamide with fludarabine (mirroring the RIC approach), has produced good results (5), particularly in CGD. Retrospective comparisons have shown a similar rate of survival with BuFlu and BuCy (6), but some physicians have been troubled by the increased MC seen with Bu Flu in contrast to BuCy.

Substituting treosulfan (Treo) for Bu is undoubtedly less toxic, with a low rate of VOD. Although the term “**reduced toxicity**” was first used to describe a Treo-based regimen (7), the combination of Treo Flu does not consistently achieve full DC and so is perhaps better labeled as RIC (See Figure 1). Treo may have a further advantage over Bu, for instance in young children with SCID, in that it does not cross the blood brain barrier and may therefore have less neurotoxic properties. On the other hand, for children with MPS1 and MLD, engraftment of brain microglial cells is dependent on Bu (at high doses) for ablation, and is superior to Treo or irradiation (8, 9). This is one example where MAC may be preferable to RIC in the non-malignant setting. Although Treo Flu can produce adequate levels of MC in children with PID, including CGD and WAS (10–12), for those with more active marrow function, the frequency of MC can be reduced by adding a third agent, thiotepa, and this 3 drug combination is usually myeloablative (13). In addition, there are diseases where more complete DC may be necessary to control some aspects of the disease, such as the auto-immunity that is seen in WAS (14) and a variety of other monogenic disease, many of which are collectively referred to as Tregopathies (15).

The combination of Flu with Melphalan was the first widely used RIC protocol in PID (16). However, its reduced myeloablation often leads to excessive MC (17), although when followed by DLI achieves excellent results in HLH (18).

## The Role of Graft-Versus-Host Disease Prophylaxis and Serotherapy

In addition to the conditioning therapy given to allow engraftment of donor cells, it is also important to prevent allogeneic T-lymphocytes from the graft reacting against the donor (or host), which causes graft-versus-host disease (GvHD).

### *Ex-vivo* T-Cell Depletion (TCD)

Some of the earliest HSCT were done using mismatched donors, often haploidentical parents. Here the risk of GvHD is so high that methods were developed to deplete the T cells from the graft, so reducing the risk of GvHD. Over the years, the techniques have become more sophisticated, from *ex-vivo* Sheep red cell rosetting and soybean lectin (19), to CD3/19 depletion and now TCRαβ-depletion (20). Although TCD also depletes cells that may be important in controlling residual leukemia, in immune deficient patients this is much less important. However, the depletion of cells that can control opportunistic infections, particularly viral infections, does contribute to morbidity and mortality after these mismatched procedures.

### *In vivo*-TCD

A variety of drugs are used to deplete T cells pre and post transplant.

### Serotherapy

Serotherapy is used, as part of conditioning therapy, to deplete function of specific *host* immune cells, which are able to reject the graft. As these agents have long half-lives, they persist after the graft is infused and so, by depleting allogeneic T-lymphocytes from the graft, can reduce GvHD (21). Two main types of “sero”therapy are in use: polyclonal preparations of anti-thymocyte globulin (ATG) from immunized rabbits or horses and a humanized monoclonal antibody targeting CD52 (Alemtuzumab). It is well-known that different brands of ATG clear at different rates, so impacting on subsequent rates of GvHD, infection and immune reconstitution (22). In the absence of any prospective head-to-head study, a retrospective study showed that immunological reconstitution was more rapid after the use of ATG (Thymoglobubulin®) 10 mg/kg in comparison to alemtuzumab (1 mg/kg), as expected from the longer half-live of the latter (23). In recent years important novel aspects beyond dosage have been considered in more detailed studies. As the therapeutic antibodies persist over several days, the timing of the administration before or even after transfusion of the transplant has an impact on the rate of GvHD and the delay in the reconstitution of donor T-lymphocytes, as shown for umbilical cord blood transplantation (24). Beyond dosage and timing, the abundance of target structures, namely T-lymphocytes has been shown to have fundamental impacts on the pharmacokinetics and the effects of ATG (25). Similar studies with Alemtuzumab, in the context of Flu MLP, have shown the expected relationship of higher peritransplant serum-levels of alemtuzumab being associated with less GvHD, slower immune reconstitution and more mixed chimerism (26). Pharmacokinetic (PK) targeting of serotherapy in conditioning is probably as desirable as PK monitoring of chemotherapy and, particularly in RIC, might achieve maximum GvM with minimal GvHD.

A variety of other **drugs** are used to inhibit T cell activation post-HSCT, such as ciclosporin and Tacrolimus. Certain chemotherapy drugs are used widely to target cells proliferating in response to allo-antigens, such as methotrexate and, most notably in the past few years, post-transplant cyclosphosphamide. The powerful effect of 2 days of cyclophosphamide given in the first few days after haploidentical HSCT has made this technique readily available to centers that do not have the laboratory skills, resources and money to support the complex procedures such as TCRαβ-depletion. Admittedly, this is more widely used in malignant disease and hematological diseases but has been used in immune deficiencies as well (27).

## Long Term Consequences of Conditioning

For long term survivors of HSCT, some long term toxicities relate to the conditioning used (see review on “long term outcome and immune function after haematopoietic stem cell transplantation for primary immunodeficiency”). In general, the more myeloablative combinations are associated with gonadal failure and infertility; whereas the RIC combinations may be associated with preservation of these important long term toxicities. Currently, long term follow up data for children treated with Treo are lacking, but early data looks promising (28).

The development of malignancy is another major long term consequence of transplant, But the majority of these appear to be secondary malignancies in patients transplanted for malignant disease, with the exception of Fanconi anemia.

## Emerging Types of Conditioning for the (Near) Future

As mentioned above, for the sick infant with newly diagnosed SCID, an unconditioned graft may be required, to achieve some T-lymphocyte function and control of infection, and then allow an older child in better shape to tolerate a conditioned graft and achieve a better functioning (donor) immune system. With the adoption of newborn screening for SCID, younger, well infants are being diagnosed earlier, at an age where the use of conventional chemotherapy is less studied and with diagnoses (such as Artemis) where the adverse consequences of chemotherapy are well-known (29). To achieve the desired outcome of conditioning with minimal toxicity, development of targeted treatment with monoclonal antibodies and other biologic agents is underway.

Preclinical studies identified CD45 (30) and CD117 (31, 32) as targets suitable for antibody mediated depletion of host hematopoietic cells. CD45, also known as the leukocyte common antigen (LCA), is expressed on all hematolymphoid cells. Because CD45 is expressed on the spectrum of primitive to mature blood cells, targeting this molecule is desirable if a single agent can safely achieve both myelo- and lymphoablation. Different forms of anti-CD45 agents have been tested including “naked” unconjugated, radio-labeled, and toxin-conjugated CD45 antibodies. Conjugation of antibodies to radio-isotopes or cytotoxic agents capitalizes on the ability of the antibodies to deliver toxic payloads to specific cell populations with the potential for more complete target cell elimination as compared to the unconjugated versions. However, the risks of the conjugates may limit the applicability of these agents to only select disorders.

A synergistic combination of two unconjugated rat anti-human CD45 antibodies has been tested in two clinical studies. A Phase 1 dose escalation study used these mAbs in patients with hematologic malignancies that received standard conditioning (33) and as part of reduced-intensity conditioning regimen for congenital immunodeficiencies, in conjunction with anti-lymphocyte agents (34). The regimen was well-tolerated and high-level donor chimerism was achieved. However, the contribution of the lytic anti-CD45 mAb treatment in the context of these other agents remains to be further clarified.

Clinical testing of radioisotope-labeled anti-CD45 mAbs are in the advanced phase in adults with myeloid disorders, and second generation agents continue to be developed (35) CD45 antibody-drug conjugates (ADC) have been shown promise in preclinical models evidenced by their ability to efficiently deplete immune cells in the periphery and HSCs in the bone marrow (36). Development of clinical grade CD45-ADC is currently underway.

Antibodies that target the cell surface receptor CD117 can also effectively clear host HSC niches. CD117 is a receptor tyrosine kinase expressed on the surface of HSC and early hematopoietic progenitors. Interaction of CD117 with its ligand, stem cell factor (SCF), provides signals necessary for HSC survival, proliferation, and differentiation. Treatment of mice with anti-CD117 mAbs results in transient depletion of HSC with return of normal blood formation within 2 weeks of antibody administration (37). This window of depletion is sufficient to permit donor cell engraftment with minimal to no off target effects.

A humanized anti-CD117 mAb, AMG 191 has been developed and is currently undergoing testing as sole conditioning for patients with SCID. Large animal studies support the safety and efficacy of this agent in depleting HSC (38). Early data from the Phase 1 dose escalation trial show proof-of-concept that targeting CD117 with biologic agents may provide a strategy for safely replacing and/or augmenting the myeloablative component of conditioning (39). Even at the lowest dose tested sustained donor HSC engraftment has been observed as evidenced by long-term myeloid chimerism, and generation of nascent B and T-lymphocytes. No toxicities related to the AMG 191 were noted, including no evidence of myelosuppression.

Pre-clinical studies have focused on increasing the niche clearing potency of the naked anti-CD117 antibodies. The combination of a second mAb directed against the macrophage checkpoint inhibitor, CD47, with an anti-CD117 mAb demonstrated marked increase in myeloablation and donor cell engraftment compared to the CD117 mAb alone (31). Other strategies which show promise include ADC-conjugates of anti-CD117 mAbs (36, 37) and the combined use of anti-CD117 with low dose radiation (40).

## Author Contributions

All authors listed have made a substantial, direct and intellectual contribution to the work, and approved it for publication.

### Conflict of Interest

The authors declare that the research was conducted in the absence of any commercial or financial relationships that could be construed as a potential conflict of interest.

## References

[1] Bacigalupo A Ballen K Rizzo D Giralt S Lazarus H Ho V. Defining the intensity of conditioning regimens: working definitions. Biol Blood Marrow Transplant. (2009) 15:1628–33. 10.1016/j.bbmt.2009.07.004 19896087 PMC2861656

[2] Horan J Wang T Haagenson M Spellman SR Dehn J Eapen M. Evaluation of HLA matching in unrelated hematopoietic stem cell transplantation for nonmalignant disorders. Blood. (2012) 120:2918–24. 10.1182/blood-2012-03-417758 22829628 PMC3466972

[3] Gatti RA Meuwissen HJ Allen HD Hong R Good RA. Immunological reconstitution of sex-linked lymphopenic immunological deficiency. Lancet. (1968) 2:1366–9. 10.1016/S0140-6736(68)92673-1 4177932

[4] Meuwissen HJ Gatti RA Terasaki PI Hong R Good RA. Treatment of lymphopenic hypogammaglobulinemia and bone-marrow aplasia by transplantation of allogeneic marrow. Crucial role of histocompatiility matching. N Engl J Med. (1969) 281:691–7. 10.1056/NEJM196909252811302 4186068

[5] Gungor T Teira P Slatter M Stussi G Stepensky P Moshous D. Reduced-intensity conditioning and HLA-matched haemopoietic stem-cell transplantation in patients with chronic granulomatous disease: a prospective multicentre study. Lancet. (2014) 383:436–48. 10.1016/S0140-6736(13)62069-3 24161820

[6] Harris AC Boelens JJ Ahn KW Fei M Abraham A Artz A Dvorak C Comparison of pediatric allogeneic transplant outcomes using myeloablative busulfan with cyclophosphamide or fludarabine. Blood Adv. (2018) 2:1198–206. 10.1182/bloodadvances.2018016956 29844205 PMC5998928

[7] Casper J Knauf W Kiefer T Wolff D Steiner B Hammer U. Treosulfan and fludarabine: a new toxicity-reduced conditioning regimen for allogeneic hematopoietic stem cell transplantation. Blood. (2004) 103:725–31. 10.1182/blood-2002-11-3615 12947008

[8] Capotondo A Milazzo R Politi LS Quattrini A Palini A Plati T. Brain conditioning is instrumental for successful microglia reconstitution following hematopoietic stem cell transplantation. Proc Natl Acad Sci USA. (2012) 109:15018–23. 10.1073/pnas.1205858109 22923692 PMC3443128

[9] Wilkinson FL Sergijenko A Langford-Smith KJ Malinowska M Wynn RF Bigger BW. Busulfan conditioning enhances engraftment of hematopoietic donor-derived cells in the brain compared with irradiation. Mol Ther. (2013) 21:868–76. 10.1038/mt.2013.29 23423338 PMC3616529

[10] Slatter MA Rao K Amrolia P Flood T Abinun M Hambleton S. Treosulfan-based conditioning regimens for hematopoietic stem cell transplantation in children with primary immunodeficiency: United Kingdom experience. Blood. (2011) 117:4367–75. 10.1182/blood-2010-10-312082 21325599

[11] Morillo-Gutierrez B Beier R Rao K Burroughs L Schulz A Ewins AM. Treosulfan-based conditioning for allogeneic HSCT in children with chronic granulomatous disease: a multicenter experience. Blood. (2016) 128:440–8. 10.1182/blood-2016-03-704015 27216217 PMC4957165

[12] Slatter MA Rao K Abd Hamid IJ Nademi Z Chiesa R Elfeky R Pearce MS. Treosulfan and fludarabine conditioning for hematopoietic stem cell transplantation in children with primary immunodeficiency: UK experience. Biol Blood Marrow Transplant. (2018) 24:529–36. 10.1016/j.bbmt.2017.11.009 29155317

[13] Shah RM Elfeky R Nademi Z Qasim W Amrolia P Chiesa R. T-cell receptor α*β*^+^ and CD19^+^ cell–depleted haploidentical and mismatched hematopoietic stem cell transplantation in primary immune deficiency. J Allergy Clin Immunol. (2018) 141:1417–26.e1. 10.1016/j.jaci.2017.07.008 28780238

[14] Moratto D Giliani S Bonfim C Mazzolari E Fischer A Ochs HD Cant AJ. Long-term outcome and lineage-specific chimerism in 194 patients with Wiskott-Aldrich syndrome treated by hematopoietic cell transplantation in the period 1980–2009: an international collaborative study. Blood. (2011) 118:1675–84. 10.1182/blood-2010-11-319376 21659547 PMC3156052

[15] Cepika AM Sato Y Liu JM Uyeda MJ Bacchetta R Roncarolo MG. Tregopathies: Monogenic diseases resulting in regulatory T-cell deficiency. J Allergy Clin Immunol. (2018) 142:1679–95. 10.1016/j.jaci.2018.10.026 30527062

[16] Rao K Amrolia PJ Jones A Cale CM Naik P King D. Improved survival after unrelated donor bone marrow transplantation in children with primary immunodeficiency using a reduced-intensity conditioning regimen. Blood. (2005) 105:879–85. 10.1182/blood-2004-03-0960 15367433

[17] Rao K Adams S Qasim W Allwood Z Worth A Silva J Lucchini G. Effect of stem cell source on long-term chimerism and event-free survival in children with primary immunodeficiency disorders after fludarabine and melphalan conditioning regimen. J Allergy Clin Immunol. (2016) 138:1152–60. 10.1016/j.jaci.2016.01.053 27241891

[18] Marsh RA Rao MB Gefen A Bellman D Mehta PA Khandelwal P. Experience with alemtuzumab, fludarabine, and melphalan reduced-intensity conditioning hematopoietic cell transplantation in patients with nonmalignant diseases reveals good outcomes and that the risk of mixed chimerism depends on underlying disease, stem cell source, and alemtuzumab regimen. Biol Blood Marrow Transplant. (2015) 21:1460–70. 10.1016/j.bbmt.2015.04.009 25865646 PMC4603747

[19] Reisner Y Kapoor N Kirkpatrick D Pollack MS Cunningham-Rundles S Dupont B. Transplantation for severe combined immunodeficiency with HLA-A,B,D,DR incompatible parental marrow cells fractionated by soybean agglutinin and sheep red blood cells. Blood. (1983) 61:341–8. 10.1182/blood.V61.2.341.bloodjournal612341 6217853

[20] Bertaina A Merli P Rutella S Pagliara D Bernardo ME Masetti R. HLA-haploidentical stem cell transplantation after removal of alphabeta^+^ T and B cells in children with nonmalignant disorders. Blood. (2014) 124:822–6. 10.1182/blood-2014-03-563817 24869942

[21] Ali R Ramdial J Algaze S Beitinjaneh A. The role of anti-thymocyte globulin or alemtuzumab-based serotherapy in the prophylaxis and management of graft-versus-host disease. Biomedicines. (2017) 5:E67. 10.3390/biomedicines5040067 29186076 PMC5744091

[22] Oostenbrink LVE Jol-van der Zijde CM Kielsen K Jansen-Hoogendijk AM Ifversen M Muller KG. Differential elimination of anti-thymocyte globulin of fresenius and genzyme impacts T-cell reconstitution after hematopoietic stem cell transplantation. Front Immunol. (2019) 10:315. 10.3389/fimmu.2019.00315 30894854 PMC6414431

[23] Willemsen L Jol-van der Zijde CM Admiraal R Putter H Jansen-Hoogendijk AM Ostaijen-Ten Dam MM. Impact of serotherapy on immune reconstitution and survival outcomes after stem cell transplantations in children: thymoglobulin versus alemtuzumab. Biol Blood Marrow Transplant. (2015) 21:473–82. 10.1016/j.bbmt.2014.11.674 25485863

[24] Lindemans CA Chiesa R Amrolia PJ Rao K Nikolajeva O de Wildt A. Impact of thymoglobulin prior to pediatric unrelated umbilical cord blood transplantation on immune reconstitution and clinical outcome. Blood. (2014) 123:126–32. 10.1182/blood-2013-05-502385 24184682

[25] Admiraal R van Kesteren C Jol-van der Zijde CM Lankester AC Bierings MB Egberts TC. Association between anti-thymocyte globulin exposure and CD4^+^ immune reconstitution in paediatric haemopoietic cell transplantation: a multicentre, retrospective pharmacodynamic cohort analysis. Lancet Haematol. (2015) 2:e194–203. 10.1016/S2352-3026(15)00045-9 26688094

[26] Marsh RA Lane A Mehta PA Neumeier L Jodele S Davies SM. Alemtuzumab levels impact acute GVHD, mixed chimerism, and lymphocyte recovery following alemtuzumab, fludarabine, and melphalan RIC HCT. Blood. (2016) 127:503–12. 10.1182/blood-2015-07-659672 26644451

[27] Klein OR Chen AR Gamper C Loeb D Zambidis E Llosa N. Alternative-donor hematopoietic stem cell transplantation with post-transplantation cyclophosphamide for nonmalignant disorders. Biol Blood Marrow Transplant. (2016) 22:895–901. 10.1016/j.bbmt.2016.02.001 26860634 PMC4898048

[28] Faraci M Diesch T Labopin M Dalissier A Lankester A Gennery A. Gonadal function after busulfan compared with treosulfan in children and adolescents undergoing allogeneic hematopoietic stem cell transplant. Biol Blood Marrow Transplant. (2019) 25:1786–91. 10.1016/j.bbmt.2019.05.005 31082473

[29] Schuetz C Neven B Dvorak CC Leroy S Ege MJ Pannicke U. SCID patients with ARTEMIS vs RAG deficiencies following HCT: increased risk of late toxicity in ARTEMIS-deficient SCID. Blood. (2014) 123:281–9. 10.1182/blood-2013-01-476432 24144642 PMC3953035

[30] Matthews DC Appelbaum FR Eary JF Hui TE Fisher DR Martin PJ. Radiolabeled anti-CD45 monoclonal antibodies target lymphohematopoietic tissue in the macaque. Blood. (1991) 78:1864–74. 1832994

[31] Chhabra A Ring AM Weiskopf K Schnorr PJ Gordon S Le AC Hematopoietic stem cell transplantation in immunocompetent hosts without radiation or chemotherapy. Sci Transl Med. (2016) 8:351ra105 10.1126/scitranslmed.aae0501 PMC6668627 27510901

[32] Czechowicz A Kraft D Weissman IL Bhattacharya D. Efficient transplantation via antibody-based clearance of hematopoietic stem cell niches. Science. (2007) 318:1296–9. 10.1126/science.1149726 18033883 PMC2527021

[33] Krance RA Kuehnle I Rill DR Mei Z Pinetta C Evans W. Hematopoietic and immunomodulatory effects of lytic CD45 monoclonal antibodies in patients with hematologic malignancy. Biol Blood Marrow Transplant. (2003) 9:273–81. 10.1053/bbmt.2003.50024 12720220

[34] Straathof KC Rao K Eyrich M Hale G Bird P Berrie E. Haemopoietic stem-cell transplantation with antibody-based minimal-intensity conditioning: a phase 1/2 study. Lancet. (2009) 374:912–20. 10.1016/S0140-6736(09)60945-4 19729196

[35] Orozco JJ Zeller J Pagel JM. Radiolabeled antibodies directed at CD45 for conditioning prior to allogeneic transplantation in acute myeloid leukemia and myelodysplastic syndrome. Ther Adv Hematol. (2012) 3:5–16. 10.1177/2040620711422265 23556108 PMC3573428

[36] Palchaudhuri R Saez B Hoggatt J Schajnovitz A Sykes DB Tate TA. Non-genotoxic conditioning for hematopoietic stem cell transplantation using a hematopoietic-cell-specific internalizing immunotoxin. Nat Biotechnol. (2016) 34:738–45. 10.1038/nbt.3584 27272386 PMC5179034

[37] Czechowicz A Palchaudhuri R Scheck A Hu Y Hoggatt J Saez B. Selective hematopoietic stem cell ablation using CD117-antibody-drug-conjugates enables safe and effective transplantation with immunity preservation. Nat Commun. (2019) 10:617. 10.1038/s41467-018-08201-x 30728354 PMC6365495

[38] Kwon HS Logan AC Chhabra A Pang WW Czechowicz A Tate K Anti-human CD117 antibody-mediated bone marrow niche clearance in non-human primates and humanized NSG mice. Blood. (2019) 133:2104–8. 10.1182/blood-2018-06-853879 30617195 PMC6509543

[39] Agarwal R Dvorak CC Prohaska S Long-Boyle J Kwon H-S Brown JM Toxicity-free hematopoietic stem cell engraftment achieved with anti-CD117 monoclonal antibody conditioning. Biol Blood Marrow Transplant. (2019) 25:S92 10.1016/j.bbmt.2018.12.172

[40] Xue X Pech NK Shelley WC Srour EF Yoder MC Dinauer MC. Antibody targeting KIT as pretransplantation conditioning in immunocompetent mice. Blood. (2010) 116:5419–22. 10.1182/blood-2010-07-295949 20813896 PMC3012550

